# Evaluation of the effectiveness of the California mosquito-borne virus surveillance & response plan, 2009–2018

**DOI:** 10.1371/journal.pntd.0010375

**Published:** 2022-05-09

**Authors:** Mary E. Danforth, Robert E. Snyder, Emma T. N. Lonstrup, Christopher M. Barker, Vicki L. Kramer

**Affiliations:** 1 Vector-Borne Disease Section, California Department of Public Health, Sacramento, California, United States of America; 2 Department of Pathology, Microbiology, and Immunology, School of Veterinary Medicine, University of California, Davis, Davis, California, United States of America; The Pennsylvania State University, UNITED STATES

## Abstract

Local vector control and public health agencies in California use the California Mosquito-Borne Virus Surveillance and Response Plan to monitor and evaluate West Nile virus (WNV) activity and guide responses to reduce the burden of WNV disease. All available data from environmental surveillance, such as the abundance and WNV infection rates in *Culex tarsalis* and the *Culex pipiens* complex mosquitoes, the numbers of dead birds, seroconversions in sentinel chickens, and ambient air temperatures, are fed into a formula to estimate the risk level and associated risk of human infections. In many other areas of the US, the vector index, based only on vector mosquito abundance and infection rates, is used by vector control programs to estimate the risk of human WNV transmission. We built models to determine the association between risk level and the number of reported symptomatic human disease cases with onset in the following three weeks to identify the essential components of the risk level and to compare California’s risk estimates to vector index. Risk level calculations based on *Cx*. *tarsalis* and *Cx*. *pipiens* complex levels were significantly associated with increased human risk, particularly when accounting for vector control area and population, and were better predictors than using vector index. Including all potential environmental components created an effective tool to estimate the risk of WNV transmission to humans in California.

## Author summary

The California Mosquito-Borne Virus Surveillance and Response Plan was designed to monitor and evaluate West Nile virus activity in the state and guide vector control and public health responses to reduce the burden of West Nile virus disease. It uses a formula to estimate the risk of human infections based on available environmental surveillance data: the abundance and infection rates in *Culex tarsalis* and the *Culex pipiens* complex mosquitoes, the numbers of dead birds, seroconversions in sentinel chickens, and/or ambient air temperatures. We evaluated the effectiveness of this risk level system and compared it to the vector index, a simpler risk estimate based only on mosquito abundance and infection rates, used by many vector control agencies inside the U.S. We found that that the California risk levels were significantly associated with increased reported human disease incidence, particularly when all potential environmental components were included, and better fit the data than vector index.

## Introduction

West Nile virus (WNV) is a flavivirus that is the leading cause of mosquito-borne disease in the United States [1,2]. Enzootic transmission of WNV is maintained in a cycle involving wild birds and mosquitoes but there is spillover that can result in human infections [3–5]. The primary mosquito vectors in California are *Culex tarsalis* and the *Cx*. *pipiens* complex [4,6], namely *Cx*. *pipiens* and *Cx*. *quinquefasciatus* and their hybrids. *Cx*. *tarsalis* is found primarily in rural, agricultural areas of the state whereas *Cx*. *pipiens* complex is found in more urban areas [7], with *Cx*. *pipiens* in the north, *Cx*. *quinquefasciatus* in the south, and both species, as well as an interspecific hybrid, found in the middle [8]. Birds in the order Passeriformes are the primary zoonotic hosts of WNV, and some species, particularly those in the family Corvidae, are regularly infected and found dead from WNV [9,10]. The complex ecology of this disease is heavily influenced by temperature, with higher temperatures resulting in increased WNV transmission due to increased mosquito abundance and a faster extrinsic incubation period [11–14].

While the majority of WNV infections are asymptomatic, 20–30% of infections result in febrile illness, with fewer than 1% of cases developing West Nile neuroinvasive disease (WNND), which has a case fatality-rate close to 10% [3,15–17]. Diagnosis and reporting of neuroinvasive disease are more complete than milder non-neuroinvasive disease due to the severity of disease [18,19]. From 2009 through 2018, 21,869 WNV disease cases were reported in the US, including 12,835 neuroinvasive cases and 1,199 deaths [20]. California reported 4,035 (18%) of all US cases, more than any other state, and disproportionate to its 12% of the nation’s population [21,22]. The majority of California’s cases have occurred in Southern California and the Central Valley [21,23].

The California Department of Public Health (CDPH), local mosquito and vector control agencies, and academic institutions in California developed an organized, comprehensive environmental surveillance program to monitor mosquito-borne viruses, beginning with mosquito testing in 1969 [24]. From its inception, the program has focused on arboviruses endemic to California, initially western equine encephalitis virus (WEEV) and St. Louis encephalitis virus (SLEV), and more recently WNV, which was first detected in the state in 2003 [25]. Vector control agencies conduct routine environmental surveillance by trapping mosquitoes, identifying and tallying the captures, and testing the primary disease vectors for the endemic viruses of WNV, SLEV, and WEEV [26]. Many agencies also collect and test dead wild birds for WNV [10]. Because chickens develop antibodies to WNV, SLEV, and WEEV after exposure but are dead end hosts and do not otherwise show signs of infection [27], many agencies maintain sentinel chicken flocks from late spring through the fall to monitor arbovirus activity in their region. Ambient air temperature, obtained from NASA’s North American Land Data Assimilation System [28,29], is also monitored. The California Mosquito-Borne Virus Surveillance and Response Plan (“Response Plan”) [30] provides guidelines for vector control agencies to estimate risk using these surveillance elements [31]. Each is assigned a weight and averaged across an area to provide an overall risk level to estimate the risk of WNV transmission in that particular area at any given time and guide response decisions. The Response Plan has been updated annually since its inception and includes a detailed description of the methodology used to calculate transmission risk [26]. The quantitative risk assessment is integrated into California’s Vectorborne Disease Surveillance (VectorSurv) system, an online data management system that supports vector-borne disease surveillance and control. VectorSurv enables local vector control agencies to enter their surveillance data and automatically estimates risk levels for any spatial area with adequate surveillance [32]. In comparison, many vector control programs outside of California rely on the vector index alone, which is a simpler measure based solely on vector abundance and mosquito infection rate, to estimate risk of human infection and trigger additional interventions [33,34].

Although previous analyses have considered the association between the Response Plan system and environmental arbovirus activity [31,35], there has never been a study examining how the risk levels are associated with reported human disease. This analysis seeks to evaluate the performance of the Response Plan for predicting human incidence of WNV disease for the years 2009 through 2018. Also, the system has been evaluated only as a whole, including all available environmental surveillance elements, so we evaluated how well human incidence was predicted when one or more environmental factors were excluded from the analysis. Finally, we sought to compare the predictive performance of the Response Plan, which incorporates many surveillance elements, compared to that of the simpler vector index, which is based only on entomological surveillance.

## Methods

### Ethics statement

Analysis of human surveillance data is a routine public health activity and therefore exempt from Institutional Review Board review and approval (Project 2020-072-CDPH).

### Human data

**Fig 1:**
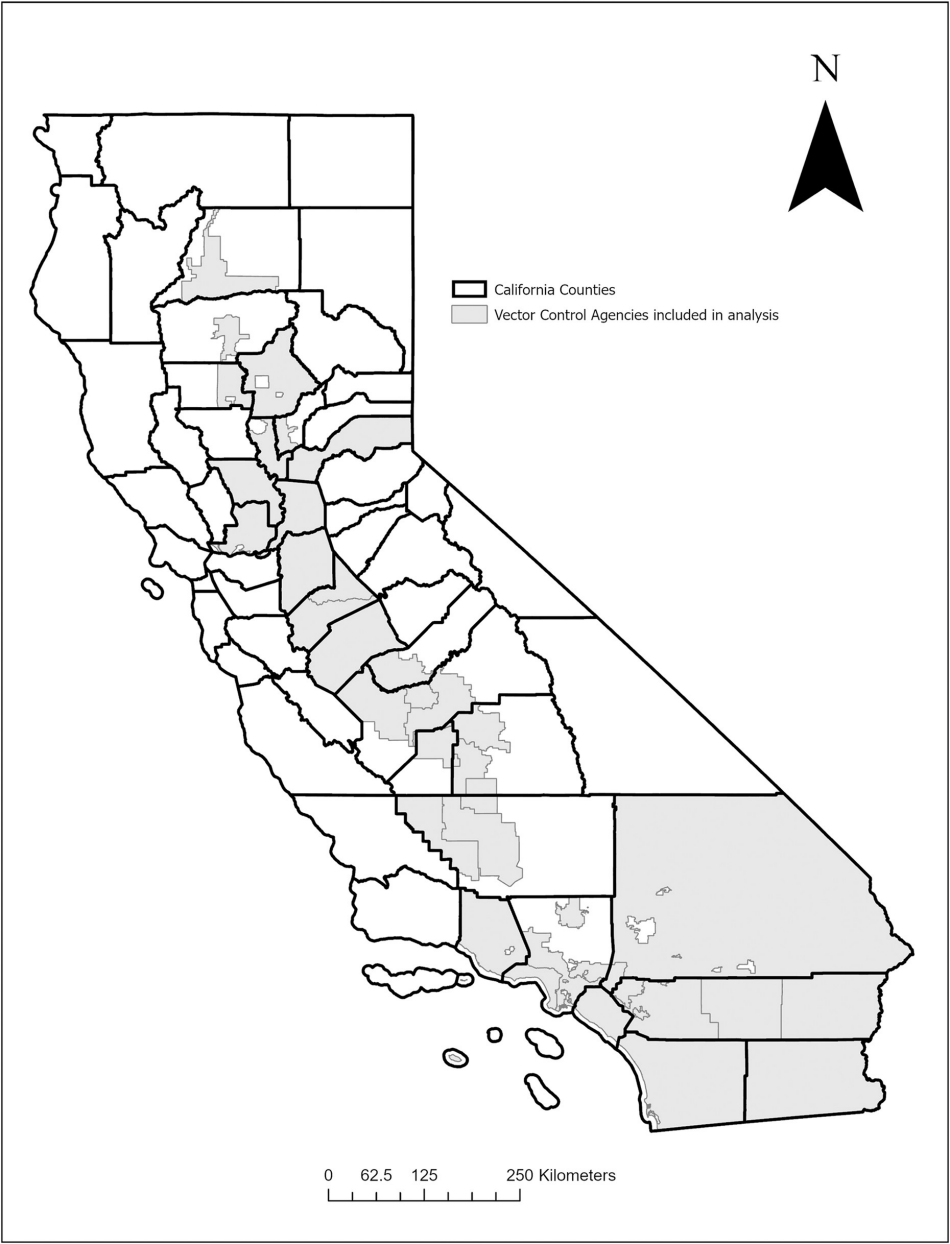
Map of California vector control agencies and counties included in analysis of effectiveness of vector index and California Response Plan, CA, 2009–2018. County data courtesy of U.S. Census Bureau TIGER/line spatial files, 2016 (https://www.census.gov/geographies/mapping-files/time-series/geo/tiger-line-file.2016.html). Vector control agency data courtesy of Mosquito and Vector Control Association of California web map, 2018, (sources: ESRI, USGS, NOAA, TomTom, U.S. Department of Commerce, U.S. Census Bureau, NPS). (https://www.arcgis.com/apps/mapviewer/index.html?webmap=604a0fe9f2b74e98a53b53d192b2ac67).

Human cases of WNV disease in California residents that meet the Council of State and Territorial Epidemiologists’ case definition [36] are reported to CDPH and subsequently to the United States Centers for Disease Control and Prevention because WNV disease is a nationally notifiable condition [37]. Asymptomatic WNV infections (such as those identified via screening of blood donors) do not fulfill the case definition and were excluded from this analysis. Other inclusion criteria for human WNV disease included disease onset between April 1 and December 31 for the years 2009 through 2018, residence in a county in the Central Valley or Southern California, and residence within the jurisdictional boundaries of a vector control agency that has at least 20,000 residents within its service area (Fig 1).

### Environmental surveillance data

Environmental surveillance data, originating from specimens collected by vector control agencies, are stored in an online data management system, the California Vectorborne Disease Surveillance Gateway (VectorSurv). Data for this project were obtained via data request #000032. We included the data elements described below for the period April 1-December 31 of each year from 2009 through 2018.

Local vector control agencies collected adult mosquitoes using a variety of traps, sorted them by species into pools of ≤50 females, and froze samples at -80°C before arbovirus testing. Mosquito abundance was calculated as the mean count of a particular species per trap-night, then divided by the prior 5-year average abundance for the same location. The University of California Davis Arbovirus Virus Research and Training (DART) laboratory conducted most arbovirus testing, although in recent years some vector control agencies began in-house testing programs. Vector control agencies that conducted their own testing passed annual proficiency panels developed by DART and reviewed by CDPH. All mosquito pools were screened for WNV, SLEV, and WEEV primarily by a multiplex real-time (TaqMan) reverse transcriptase-polymerase chain reaction (RT-qPCR) or singleplex RT-qPCR, although a few agencies used rapid commercial antigen-capture assay (RAMP, Rapid Analyte Measurement Platform, Response Biomedical Co., Vancouver, BC, Canada). Mosquito infection rates were calculated per 1,000 mosquitoes. CDPH was notified of dead birds by the public via a WNV call center, an online reporting system, and reports that came through vector control agencies. Carcasses that fulfilled collection criteria and were in acceptable condition were collected by vector control agencies and submitted to DART or tested in-house. Tissue samples and oral swabs were tested by RT-qPCR, RAMP, or VecTOR/VecTest (Medical Analysis Systems, Inc., Camarillo, CA). Dead birds were reported as the number of positive birds in a broad or specific region during the prior 3-month period. Sentinel chickens were obtained each spring by participating vector control agencies and tested to ensure that they had no antibodies for WNV, SLEV, or WEEV. Flocks of 6 to 10 chickens were established at locations specified by vector control agencies and bled biweekly by comb prick from April through November, then tested for WNV antibodies at CDPH. Chicken surveillance was reported as the number of seroconversions within a flock in a broad or specific region. The temperature data were obtained from NASA’s North American Land Data Assimilation System [28,29].

### West-Nile virus risk assessment

Each environmental variable was assigned an ordinal number from 1 through 5 based on the observations within a 2-week window, as defined in the risk assessment model in the California Mosquito-Borne Virus Surveillance and Response Plan (Table 1) [26]. The biweekly interval was reset and recalculated every week (e.g. the value from week 19 represented data from weeks 18 and 19, while the week 20 value represented data from weeks 19 and 20). Mosquito abundance and infection rate were calculated separately for *Cx*. *tarsalis* and *Cx*. *pipiens* complex mosquitoes. For each 2-week interval within a given geographic area that had observations for temperature and at least one other environmental component, all available environmental risk values were then averaged to calculate the overall risk level for *Cx*. *tarsalis* and *Cx*. *pipiens* complex mosquitoes, ranging between 1 and 5. Risk levels between 1.0 and 2.5 are considered Normal Season, 2.6 to 4.0 Emergency Planning, and 4.1 to 5.0 Epidemic Conditions.

**Table 1:** Mosquito-Borne Virus Risk Assessment for West Nile virus, from the California Mosquito-Borne Virus Surveillance & Response Plan, by California Department of Public Health, Mosquito & Vector Control Association of California, and University of California, published May 2021.

WNV Surveillance Factor	Assessment Value	Benchmark
**Temperature Conditions**	1	Avg. daily temperature during prior 2 weeks ≤56°F
	2	Avg. daily temperature during prior 2 weeks 57–65°F
	3	Avg. daily temperature during prior 2 weeks 66–72°F
	4	Avg. daily temperature during prior 2 weeks 73–79°F
	5	Avg. daily temperature during prior 2 weeks >79°F
**Relative abundance of adult female *Culex tarsalis* and *Cx*. *pipiens* complex mosquitoes**	1	Vector abundance well below average (≤50%)
	2	Vector abundance below average (51–90%)
	3	Vector abundance average (91–150%)
	4	Vector abundance above average (151–300%)
	5	Vector abundance well above average (>300%)
**Virus infection rate in *Cx*. *tarsalis* and *Cx*. *pipiens* complex mosquitoes** (MIR = mosquito infection rate per 1,000 mosquitoes)	1	MIR = 0
	2	MIR = 0.1–1.0
	3	MIR = 1.1–2.0
	4	MIR = 2.1–5.0
	5	MIR>5
**Sentinel chicken seroconversion**	1	No seroconversions in broad region
	2	≥1 seroconversions in broad region
	3	1 or 2 seroconversions in specific region
	4	≥2 seroconversions in a single flock or 2 flocks with 1 or 2 seroconversions in specific region
	5	≥2 seroconversions per flock in multiple flocks in specific region
**Dead bird infection**	1	No positive dead birds in broad region
	2	≥1 positive dead birds in broad region
	3	1 positive dead bird in specific region
	4	2–5 positive dead birds in specific region
	5	>5 positive dead birds in specific region

### Statistical analysis

Data were analyzed in R Statistical Software version 4.0.2 [38], using package glmmTMB version 1.0.2.1 [39] and package DHARMa version 0.4.4 [40]. Because several vector control agencies span multiple counties, all data were aggregated spatially within each unique combination of county and agency, hereafter called vector control areas (VCAs). Each VCA operates independently, making decisions on the extent of their surveillance, which could impact when and where WNV was detected. The outcome, measured as the number of human cases of WNV disease that occurred in a 3-week period within each VCA, was analyzed by negative binomial regression. To compare the predictive value of the full suite of environmental surveillance elements versus reduced subsets typical of local programs with fewer resources, overall risk levels were also calculated by systematically excluding one or more components from calculations. Vector index was calculated by multiplying the mean number of mosquitoes captured per trap night by the mosquito infection rate per 1,000 mosquitoes, by species, for the VCA. Other elements included in all models were: 1) random intercepts for each VCA to allow for variation in baseline WNV disease incidence and differences in surveillance and control practices among VCAs, and 2) the log of the 2017 population [41] as an offset.

To determine how accurately the Response Plan predicts human cases, models were stratified by *Cx*. *tarsalis* and *Cx*. *pipiens* complex. To determine which components were essential to the Response Plan, data were used for time periods and locations which had all five risk components from agencies that consistently operate and collect data for sentinel chicken and dead bird programs. Risk levels were compared to the vector index using data from time periods and locations which had both abundance and infection rate data for both *Cx*. *tarsalis* and *Cx*. *pipiens* complex.

All models were compared using the Akaike information criterion (AIC), where the lowest AIC value indicates the best-fitting model, though AIC differences ≤2 are not considered significant [42]. The best-fitting model for research question was then analyzed for spatial autocorrelation by Moran’s I, using distance measurements based on the centroid for each VCA. To reduce the influence of multiple time points on spatial autocorrelation, all longitude values were shifted by 10,000 km each biweekly interval.

## Results

**Fig 2:**
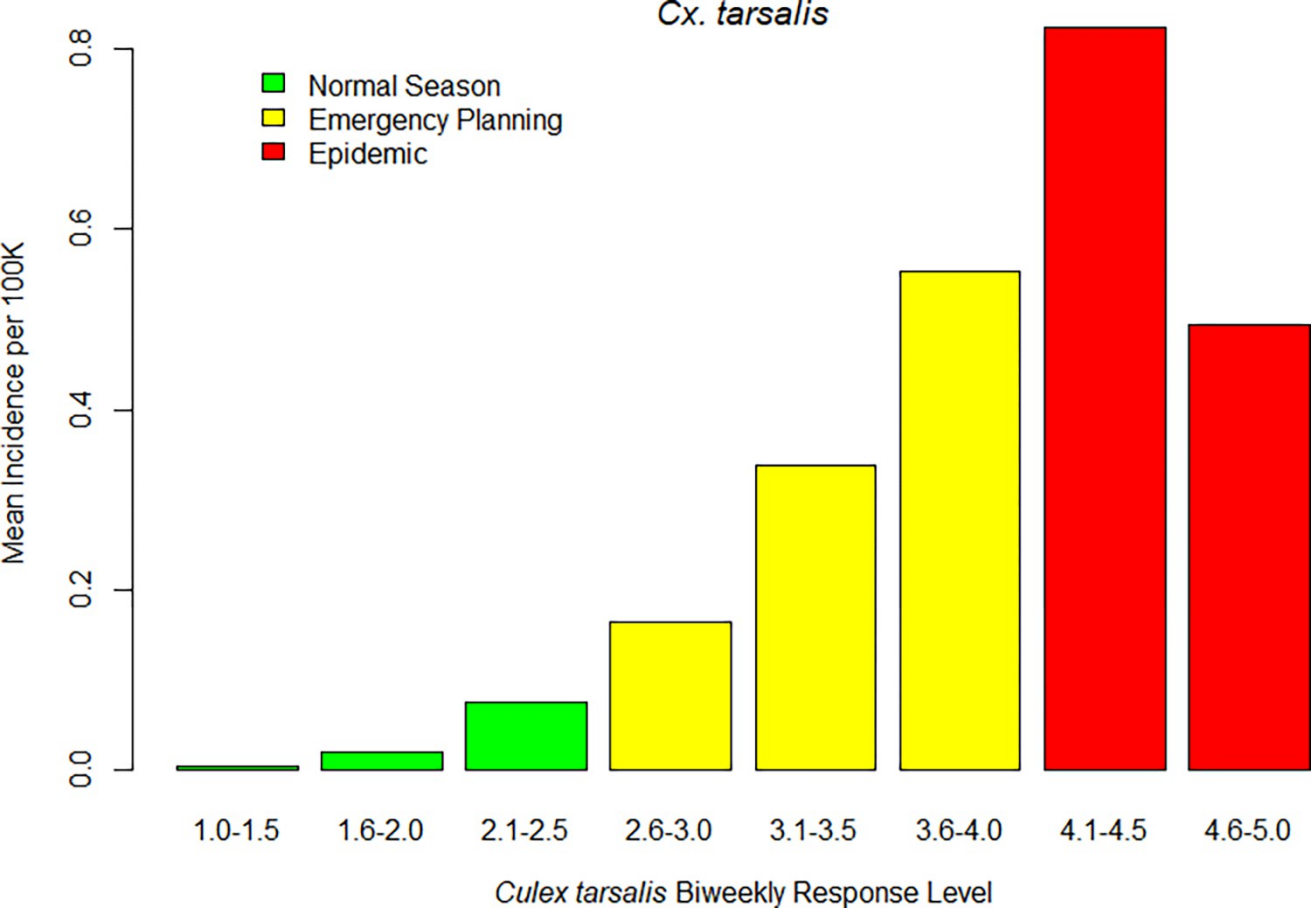
Observed human WNV disease incidence in 3-week periods versus antecedent biweekly overall risk levels, based on temperature, abundance and at least 1 infection indicator, for *Cx*. *tarsalis* in California, 2009–2018.

**Fig 3:**
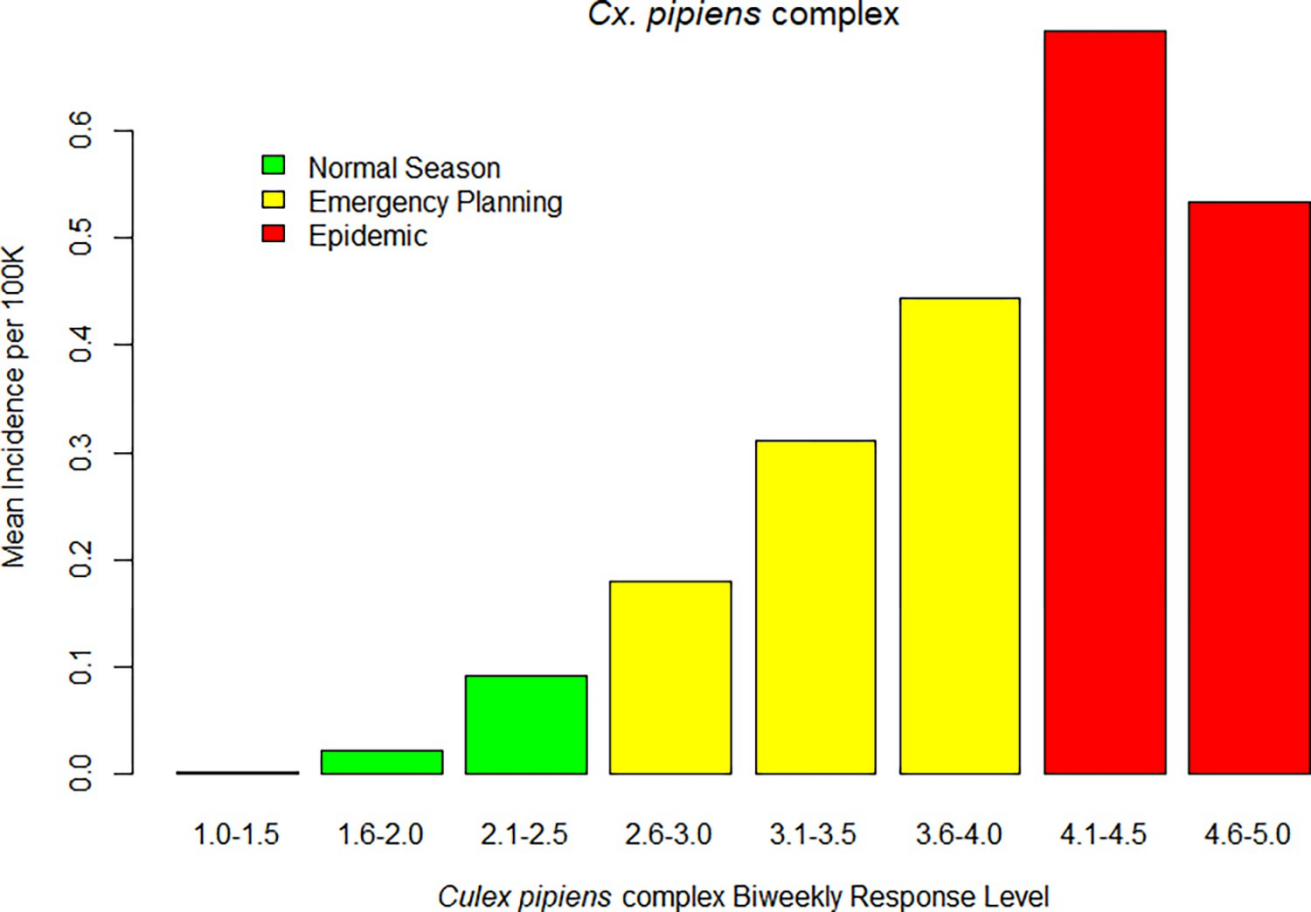
Observed human WNV disease incidence in 3-week periods versus antecedent biweekly overall risk levels, based on temperature, abundance and at least 1 infection indicator, for the *Cx*. *pipiens* complex in California, 2009–2018.

During the study period from 2009 through 2018, 4,123 cases of WNV disease in humans were reported in California. Of those, 3,614 (88%) cases met the inclusion criteria of residing in a Central Valley or Southern California county, within a vector control agency that serves ≥20,000 people, and with symptom onset between April 1 and December 31. There were 15,210 biweekly time periods within VCAs with sufficient environmental surveillance data to calculate risk levels. Observed disease incidence per 100,000 individuals generally increased as the observed overall *Cx*. *tarsalis* and *Cx*. *pipiens* complex risk levels increased (Figs 2 and 3).

**Fig 4:**
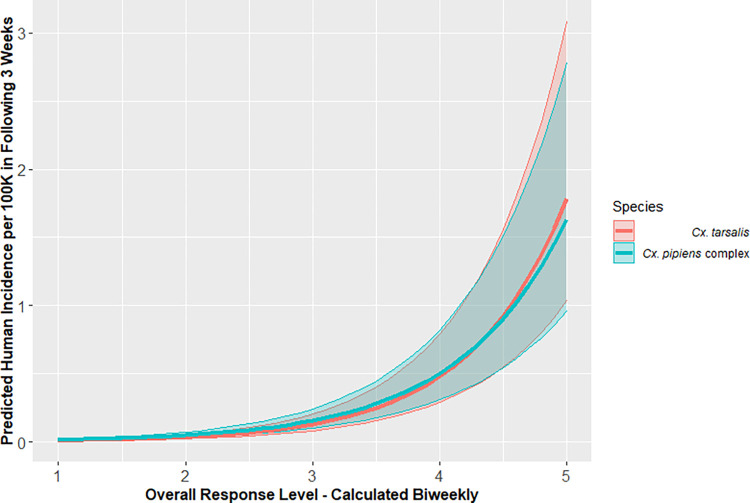
Predicted incidence of human WNV disease with 95% confidence interval during 3-week periods over the range of biweekly overall risk levels for California, 2009–2018.

Records from 36 VCAs had data for both *Cx*. *tarsalis* and *Cx*. *pipiens* complex mosquitoes, resulting in 12,364 observations (S1 Table). When comparing models, including population as an offset and agency as a random effect produced the models with the lowest AIC for predicting human disease incidence using both the *Cx*. *tarsalis* and *Cx*. *pipiens* complex risk levels (Tables 2 and S2). When controlling for human population and VCA, the incidence of human disease increased 274% for every unit increase in *Cx*. *pipiens* complex overall risk level, a risk ratio of 3.74 (95% CI: 3.58–3.90) and 224% for every unit increase in *Cx*. *tarsalis* overall risk level, a risk ratio of 3.24 (95% CI: 3.11–3.38). Though models using the *Cx*. *pipiens* complex risk level had lower AIC values than those with *Cx*. *tarsalis* risk level, their 95% CI had nearly perfect congruence (Fig 4). Both models had significant distance-based autocorrelation (p<0.01). All subsequent comparisons were made using models that included an environmental predictor (risk level or vector index), population as an offset, and VCA as a random effect.

**Table 2:** Comparison of models that predict human WNV disease occurrence, CA, 2009–2018, ranked by Akaike Information Criterion (AIC). Lower AIC values indicate better fit.

Model Components	ΔAIC
**Overall *Cx*. *pipiens* complex risk level *+* offset(log(population)) + (1|VCA)**	**(referent)**
Overall *Cx*. *pipiens* complex risk level + (1|VCA)	7.6
**Overall *Cx*. *tarsalis* risk level + offset(log(population)) + (1|VCA)**	**61.2**
Overall *Cx*. *tarsalis* risk level + (1|VCA)	677.9
Overall *Cx*. *pipiens* complex risk level + offset(log(population))	2,051.3
Overall *Cx*. *pipiens* complex risk level	2,118.0
Overall *Cx*. *tarsalis* risk level + offset(log(population))	2,489.0
Overall *Cx*. *tarsalis* risk level	2,842.8

**Fig 5:**
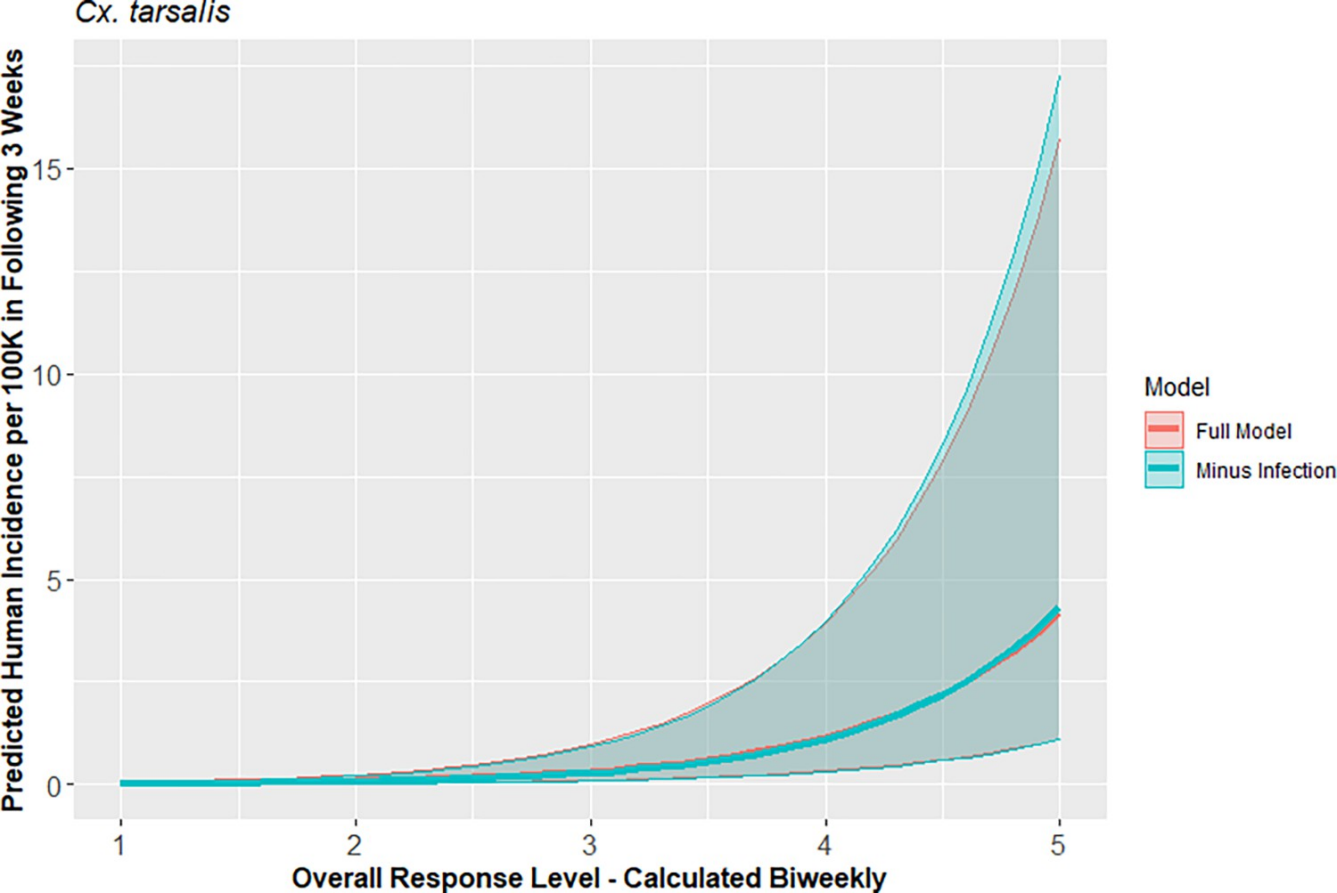
Comparison of predicted human WNV incidence using *Cx*. *tarsalis*-based response level models including all environmental components vs best drop-out model, with 95% confidence intervals, in California, 2009–2018.

**Fig 6:**
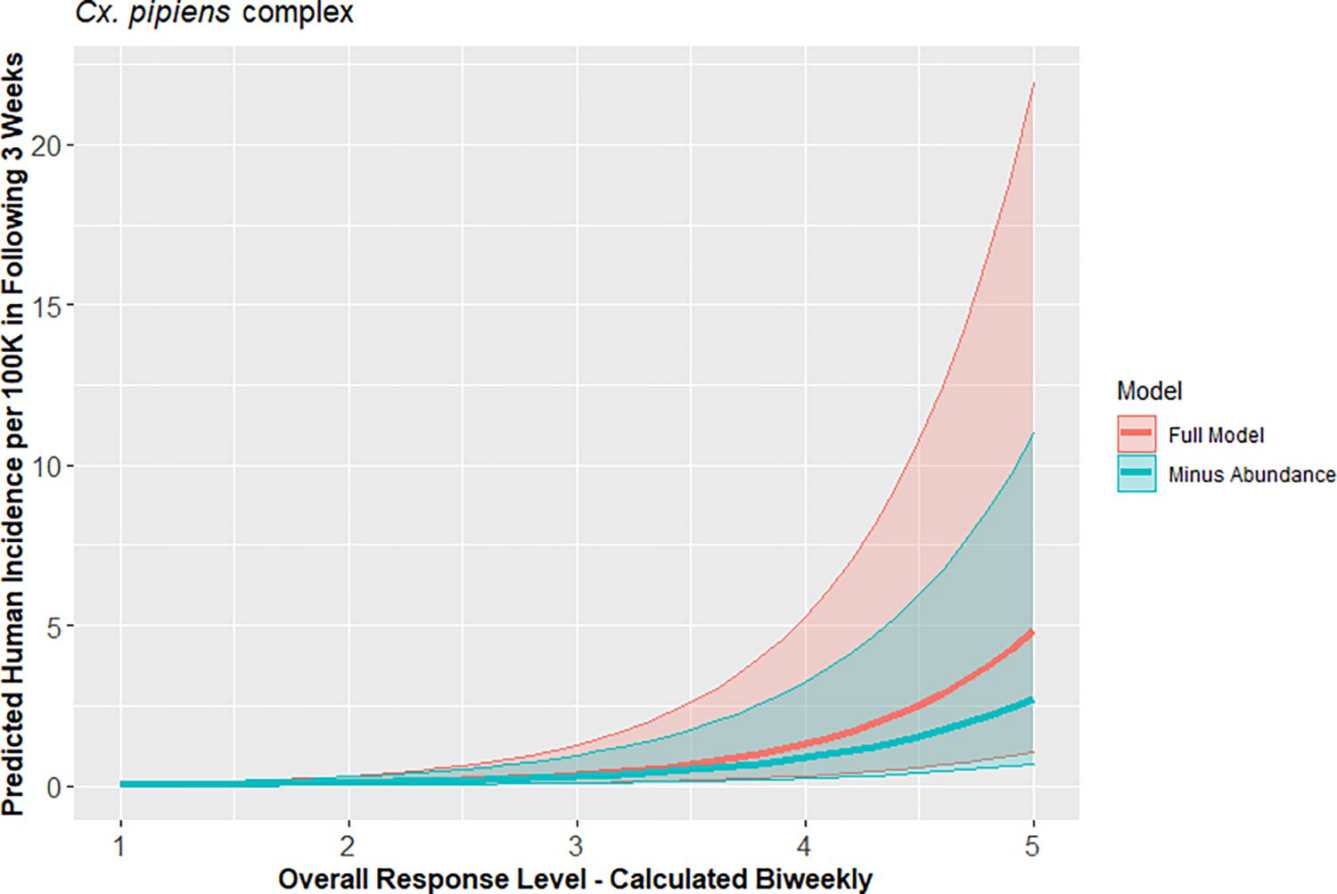
Comparison of predicted human WNV incidence using *Cx*. *pipiens* complex-based response level models including all environmental components vs best drop-out model, with 95% confidence intervals, in California, 2009–2018.

Seven VCAs consistently operated sentinel chicken and dead bird surveillance programs during this time period; these vector control agencies covered all or most of Butte, Los Angeles, Placer, Sacramento, Sutter, Yolo, and Yuba counties. Collectively, these agencies had 1,176 observations of risk levels when all five environmental components were available, including abundance and infection data for both *Culex* species. Risk levels for *Cx*. *tarsalis* and *Cx*. *pipiens* complex calculated using all available components were on average better than risk levels that omitted components (Tables 3 and S3). Interestingly, the *Cx*. *tarsalis* risk level was a better predictor of human incidence when mosquito infection rate was omitted. Similarly, predicting human incidence by *Cx*. *pipiens* complex response level was slightly improved by omitting mosquito abundance. While AIC was slightly lower than the full model for *Cx*. *pipiens* complex when only temperature and mosquito infection rate were included, the difference was not significant. Although response level calculations that omitted one variable (i.e., drop out models) were technically stronger than the response levels that included all components for both *Cx*. *tarsalis* and *Cx*. *pipiens* complex based on AIC, the 95% CI for the full and drop out models did not differ significantly in their ability to predict human incidence (Figs 5 and 6). The best two models for *Cx*. *tarsalis* and *Cx*. *pipiens* complex had significant distance-based autocorrelation (p<0.01).

**Table 3:** Comparisons of models based on different calculations for risk level, ranked by Akaike Information (AIC).

Components Included in Risk Level Calculation					*Cx*. *pipiens* complex models	*Cx*. *tarsalis* models
Temperature	Abundance	Infection	Dead Birds	Sentinel Chickens	ΔAIC	ΔAIC
*Yes*	*Yes*	*Yes*	*Yes*	*Yes*	*(referent)*	*(referent)*
Yes	--	Yes	Yes	Yes	-11.3*	8.1
Yes	Yes	--	Yes	Yes	31.2	-22.7*
Yes	Yes	Yes	--	Yes	13.4	18.6
Yes	Yes	Yes	Yes	--	16.6	18.1
--	Yes	Yes	Yes	Yes	17.7	18.6
Yes	Yes	Yes	--	--	39.8	42.4
Yes	--	Yes	--	--	-1.9	42.3
--	Yes	Yes	--	--	73.9	73.4

* indicates a significant improvement in model fit compared to the referent all-elements model.

**Fig 7:**
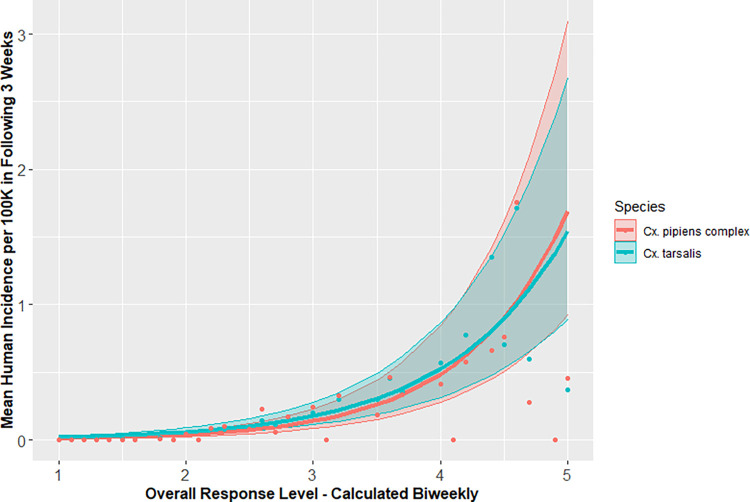
Predicted human WNV incidence estimated using California Mosquito-Borne Virus Surveillance & Response Plan, with 95% confidence intervals, with observed mean human WNV incidence (as points), in California, 2009–2018.

**Fig 8:**
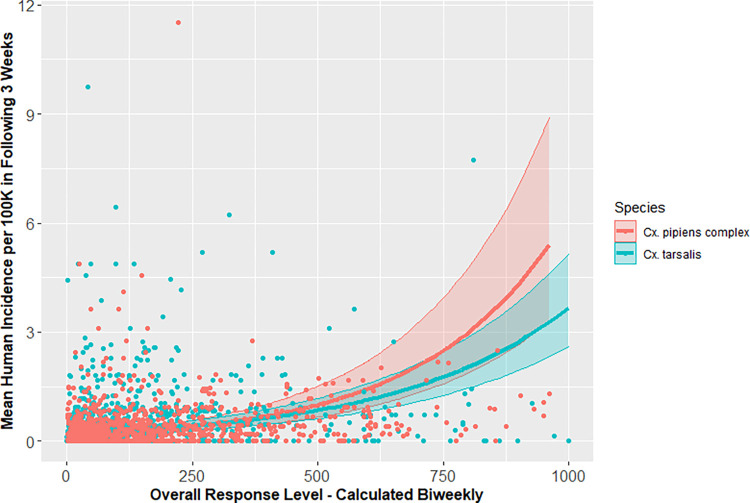
Predicted human WNV incidence estimated using vector index, with 95% confidence intervals, with observed mean human WNV incidence (as points), in California, 2009–2018.

Sufficient data were available to calculate vector index values from 34 VCAs, yielding observed values for 6,600 biweekly periods, the same time scale as response levels. Most observations (56.4%) of vector index for *Cx*. *pipiens* complex were 0, with a mean of 77.2 (range: 0–25,954), whereas 65.1% of observations of vector index for *Cx*. *tarsalis* were 0, with a mean of 40.5 (range: 0–2,709). To reduce the influence of outliers, the data was subset to vector index observations ≤1,000, which left 98% of observations for both *Cx*. *pipiens* complex (1,528/1,561) and *Cx*. *tarsalis* (1,307/1,330). Models based on a response level had a lower AIC than the associated model based on vector index for the same species. The AIC of the regression model based on *Cx*. *pipiens* complex vector index was 1,336 higher than that which was based on response level, while the ΔAIC for *Cx*. *tarsalis* vector index model was 1,421 higher than the response level model, indicating that the response level was a better predictor of human disease incidence overall compared to vector index (Figs 7 and 8 and S4 Table). All four models had significant distance-based autocorrelation by the Moran’s I test (p<0.01).

## Discussion

We found that the current California arbovirus surveillance system was positively associated with reported human WNV disease incidence and that relationship was improved after accounting for population size and baseline variation among local vector control areas (VCAs). The latter element encompasses geographic, ecological, and administrative differences across various regions of California. There were no significant differences in predicted human disease incidence when using environmental indicators based on *Cx*. *tarsalis* or *Cx*. *pipiens* complex vectors. Although there were models that omitted specific elements that more accurately predicted human disease incidence than those that included all possible environmental elements, namely omitting *Cx*. *pipiens* complex mosquito abundance or *Cx*. *tarsalis* mosquito infection, there was no significant difference in model fit when comparing these to the full model. Including all possible environmental elements in Response Plan risk calculations predicted human WNV disease incidence and also simplifies both data entry and response decisions for vector control agencies. The Response Plan improved predictions of human incidence compared to the vector index for both *Cx*. *tarsalis* and *Cx*. *pipiens* complex.

There have been numerous studies investigating WNV risk in California, seeking to identify important environmental predictors. Hartley et. al. successfully used two components from the California Vectorborne Disease Surveillance System, temperature and mosquito abundance, to predict a third component, sentinel chicken seroconversions, as a measure of virus transmission [43]. The Dynamic Continuous-Area Space-Time (DYCAST) system used dead bird reports to identify areas at high risk of WNV transmission to humans, with an 81% sensitivity and 91% specificity during an epidemic year, though predictive performance declined in non-epidemic years [44]. Kwan et al. compared how well the Response Plan, vector index, and DYCAST predicted human WNV cases in Los Angeles, California and found that the including surveillance data from bird, mosquito, and temperature sources led to the best risk assessment model for human WNV cases [45].

When identifying important components of the Response Plan, we used data only from VCAs that had robust dead bird and sentinel chicken programs and had available data monitoring all five possible environmental components. Since data for all Response Plan elements are not collected by most VCAs, only temperature and one other environmental parameter are required to estimate risk level. However, our models showed the additional value when agencies were able to incorporate dead birds and sentinel chickens because they improved predictions of human disease risk. Whenever possible, VCAs should consider including these components, as well as mosquito and temperature data, when feasible in their arbovirus surveillance system. A previous analysis found that in areas with susceptible bird species, surveillance systems based on dead birds reported by the public along with strategic mosquito and sentinel flock monitoring was effective and efficient in estimating risk in terms of both cost and effect [46].

The vector index is used in many US states for estimating risk of human disease caused by WNV and other arboviruses [33,34,47,48]. Our models showed a significant positive relationship between human incidence and vector index (*P* < 0.001), when accounting for population and VCA. However, models that used California’s risk level system had a better fit, indicating that they better explained the variability in WNV disease incidence. This is likely attributable to the additional elements included in the Response Plan: temperature, which directly influences viral replication and the rate of transmission, and dead birds and sentinel chickens, which are effective indicators of virus transmission. This study’s primary focus was on the predictive performance of the California response plan’s risk model, including the full range of surveillance elements or subsets thereof. We compared the California risk model’s estimates to the vector index alone as a typical basis for vector control action thresholds, and future studies would be useful to evaluate other ways to synthesize available environmental and surveillance data to predict human disease risk.

One limitation of this study is that our estimates of human disease incidence were based on reported cases of WNV; however, WNV is a vastly underreported disease. WNND is most likely to be diagnosed and reported, due to its severity of disease, but it is estimated that fewer than 5% of non-neuroinvasive disease cases are diagnosed and reported [2,19]. For every neuroinvasive disease case, there are an estimated 30–70 non-neuroinvasive cases, which means the true annual incidence of WNV in California is between 19 and 45 cases per 100,000 people [21], considerably higher than the observed incidence of 1.1 cases per 100,000 during this time-period [23]. WNV reporting is also likely influenced by additional factors such as access to healthcare and socio-economic status, which could lead to underreporting from some areas. In addition, this analysis focused exclusively on the Central Valley and Southern California, areas with high levels of endemic WNV transmission and correspondingly higher burdens of WNV disease. Excluding areas with lower levels of WNV activity eliminated some data at the lower end of the risk levels. However, by extending the study period to slightly before and after the season when most human WNV infections occur, we have accounted for the full range of conditions under which human cases were likely to occur and those during which transmission risk was low. Human infectious disease cases are reported based on the jurisdiction within which the case-patients reside and does not take into consideration the possibility that these individuals may spend considerable amounts of time outside that jurisdiction for work and/or recreation.

There was spatial autocorrelation within each of our models which limits the interpretability of our study. Public health surveillance data is not randomly collected; VCAs monitor areas where there have been previously high levels of virus activity. In addition, VCAS may increase mosquito trapping and testing in areas where there have been other indicators of viral activity, including dead birds, human cases, and even equine cases. VCAs are often within close proximity to one another and have similar ecologies, so the risk within one VCA can influence the risk within another.

This study aimed to compare the predictive performance of the Response Plan’s risk assessment and the vector index in a relative sense to identify the surveillance components that were most predictive of disease incidence. Based on these analyses, it is not possible to declare that the incidence predicted by a given model is “accurate,” only that it was more accurate than other models considered. Further study would be needed to evaluate predictive performance of the Response Plan and vector index using operationally relevant measures of accuracy, such as the sensitivity or specificity of various action thresholds that could be used as triggers for public health or vector control actions. All models treated human disease risk versus environmental surveillance factors as a simple logarithmic relationship: as response level increased, so did human risk. However, our observed data shows a slight but consistent decline in human incidence following time periods with the highest response level for both *Cx*. *tarsalis* and *Cx*. *pipiens* complex. This may be due to activities conducted by local vector control agencies in response to these elevated levels which are not accounted for in this analysis, such as the use of adult mosquito control products (adulticides) at the highest response levels, thus lowering human disease risk. As there are no treatments or vaccines available for WNV [49,50], the use of adulticides is one the strongest tools available to combat human disease. During a season with epidemic WNV disease and environmental conditions, Sacramento-Yolo Mosquito and Vector Control spent $700,000 to provide emergency vector control, including adulticiding, covering the county’s 2,570 km^2^ [51]. With an estimated cost of $33,000 to treat each WNV neuroinvasive disease case, it was more cost effective to adulticide than treat patients if only 15 cases were prevented [52]. More recent estimates from California found a median charge for treating a WNND case of $142,321, making adulticiding an even more cost-effective tool for mitigation of human disease [53].

Building a WNV disease surveillance program that includes the monitoring of vertebrate hosts and temperature, in addition to mosquito abundance and infection rates, yields a robust system for predicting when and where human cases are most likely to occur. The VectorSurv system then automatically analyzes the data and generates risk levels that are easily interpretable, and each state has the ability to adjust risk thresholds for each environmental surveillance component based on their own analysis [54]. This automation of the Response Plan’s risk assessment allows California’s local vector control and public health agencies to allocate resources optimally to reduce human disease risk, including public education on personal protection and enhanced mosquito surveillance and control efforts.

## Supporting information

S1 Table Counties and vector control agencies included in these analyses. (DOCX)

S2 Table Comparison of coefficients from predictive models of human WNV disease occurrence, CA, 2009–2018, ranked by Akaike Information Criterion (AIC). Lower AIC values indicate better fit.
(DOCX)

S3 Table Comparisons of coefficients based on models for different calculations for risk level, ranked by Akaike Information (AIC). (DOCX)

S4 Table Comparison of coefficients from models to predict human WNV disease occurrence, CA, 2009–2018 by Response Plan and vector index, ranked by Akaike Information Criterion (AIC). Lower AIC values indicate better fit.
(DOCX)
