# Supplementary material for: Evaluation of the effectiveness of the California mosquito-borne virus surveillance & response plan, 2009–2018
Source: PLoS Negl Trop Dis. 2022 May 9;16(5):e0010375. doi: 10.1371/journal.pntd.0010375 (PMC9119623; doi:10.1371/journal.pntd.0010375)
Supplement: S1 Table — (DOCX) [file pntd.0010375.s001.docx]

**S1 Table**: Counties and vector control agencies included in these analyses.

| **County** | **Agency** |
| --- | --- |
| Butte | Butte County Mosquito and Vector Control District |
| Fresno | Consolidated Mosquito Abatement District |
| Fresno | Fresno Mosquito & Vector Control District |
| Fresno | Fresno Westside Mosquito Abatement District |
| Glenn | Glenn County Mosquito and Vector Control District |
| Imperial | Imperial County Vector Control |
| Kern | Delano Mosquito Abatement District |
| Kern | Kern Mosquito & Vector Control District |
| Kern | West Side Mosquito and Vector Control District |
| Kings | Kings Mosquito Abatement District |
| Los Angeles | Antelope Valley Mosquito and Vector Control District |
| Los Angeles | Greater Los Angeles County Vector Control District |
| Los Angeles | Los Angeles County West Vector and Vector-Borne Disease Control District |
| Los Angeles | Long Beach Vector Control Program |
| Los Angeles | San Gabriel Valley Mosquito and Vector Control District |
| Madera | Madera County Mosquito and Vector Control District |
| Merced | Merced County Mosquito Abatement District |
| Orange | Orange County Mosquito and Vector Control District |
| Placer | Placer Mosquito and Vector Control District |
| Riverside | Coachella Valley Mosquito and Vector Control District |
| Riverside | Northwest Mosquito and Vector Control District |
| Riverside | Riverside County Environmental Health Vector Control Program |
| Sacramento | Sacramento-Yolo Mosquito and Vector Control Program |
| San Bernardino | San Bernardino County Mosquito and Vector Control Program |
| San Bernardino | West Valley Mosquito and Vector Control District |
| San Diego | San Diego County Department of Environmental Health VCP |
| San Joaquin | San Joaquin County Mosquito and Vector Control |
| Shasta | Shasta Mosquito and Vector Control District |
| Solano | Solano County Mosquito Abatement District |
| Stanislaus | East Side Mosquito Abatement District |
| Stanislaus | Turlock Mosquito Abatement District |
| Sutter | Sutter-Yuba Mosquito and Vector Control District |
| Tehama | Tehama County Mosquito and Vector Control District |
| Tulare | Delano Mosquito Abatement District |
| Tulare | Delta Vector Control District |
| Tulare | Tulare Mosquito Abatement District |
| Ventura | Ventura County Environmental Health Division |
| Yolo | Sacramento-Yolo Mosquito and Vector Control Program |
| Yuba | Sutter-Yuba Mosquito and Vector Control District |
