# Supplementary material for: Evaluation of the effectiveness of the California mosquito-borne virus surveillance & response plan, 2009–2018
Source: PLoS Negl Trop Dis. 2022 May 9;16(5):e0010375. doi: 10.1371/journal.pntd.0010375 (PMC9119623; doi:10.1371/journal.pntd.0010375)
Supplement: S2 Table — Lower AIC values indicate better fit. (DOCX) [file pntd.0010375.s002.docx]

**S2 Table**: Comparison of coefficients from predictive models of human WNV disease occurrence, CA, 2009-2018, ranked by Akaike Information Criterion (AIC). Lower AIC values indicate better fit.

| **Model Components** | **Intercept (p-value)** | **Slope**  **(p-value)** | **ΔAIC** |
| --- | --- | --- | --- |
| *Overall Cx. pipiens complex risk level + offset(log(population)) + (1\|VCA)* | *-17.52 (<0.05)* | *1.32 (<0.05)* | *(referent)* |
| Overall *Cx. pipiens* complex risk level + (1\|VCA) | -4.80 (<0.05) | 1.32 (<0.05) | 7.6 |
| *Overall Cx. tarsalis risk level + offset(log(population)) + (1\|VCA)* | *-16.90 (<0.05)* | *1.18 (<0.05)* | *61.2* |
| Overall *Cx. tarsalis* risk level + (1\|VCA) | -4.18 (<0.05) | 1.17 (<0.05) | 677.9 |
| Overall *Cx. pipiens* complex risk level + offset(log(population)) | -18.14 (<0.05) | 1.34 (<0.05) | 2,051.3 |
| Overall *Cx. pipiens* complex risk level | -17.57 (<0.05) | 1.27 (<0.05) | 2,118.0 |
| Overall *Cx. tarsalis* risk level + offset(log(population)) | -3.22 (<0.05) | 0.98 (<0.05) | 2,489.0 |
| Overall *Cx. tarsalis* risk level | -2.43 (<0.05) | 0.76 (<0.05) | 2,842.8 |
