# Supplementary material for: Evaluation of the effectiveness of the California mosquito-borne virus surveillance & response plan, 2009–2018
Source: PLoS Negl Trop Dis. 2022 May 9;16(5):e0010375. doi: 10.1371/journal.pntd.0010375 (PMC9119623; doi:10.1371/journal.pntd.0010375)
Supplement: S3 Table — (DOCX) [file pntd.0010375.s003.docx]

**S3 Table**. Comparisons of coefficients based on models for different calculations for risk level, ranked by Akaike Information (AIC).

| **Components Included in Risk Level Calculation** | | | | | ***Cx. pipiens* complex models** | | | ***Cx. tarsalis* models** | | |
| --- | --- | --- | --- | --- | --- | --- | --- | --- | --- | --- |
| **Temp.** | **Abun.** | **Inf.** | **Dead Birds** | **Chic-kens** | **Intercept**  **(p-value)** | **Slope**  **(p-value)** | **ΔAIC** | **Intercept**  **(p-value)** | **Slope**  **(p-value)** | **ΔAIC** |
| *Yes* | *Yes* | *Yes* | *Yes* | *Yes* | *-16.58*  *(<0.05)* | *1.34*  *(<0.05)* | *(ref)* | *-16.57 (<0.05)* | *1.30*  *(<0.05)* | *(ref)* |
| Yes | -- | Yes | Yes | Yes | -16.23  (<0.05) | 1.15  (<0.05) | -11.3* | -15.82  (<0.05) | 1.04  (<0.05) | 8.1 |
| Yes | Yes | -- | Yes | Yes | -16.73  (<0.05) | 1.36  (<0.05) | 31.2 | -16.92  (<0.05) | 1.38  (<0.05) | -22.7* |
| Yes | Yes | Yes | -- | Yes | -16.30  (<0.05) | 1.29  (<0.05) | 13.4 | -16.27  (<0.05) | 1.25  (<0.05) | 18.6 |
| Yes | Yes | Yes | Yes | -- | -16.64  (<0.05) | 1.32  (<0.05) | 16.6 | -16.40  (<0.05) | 1.23  (<0.05) | 18.1 |
| -- | Yes | Yes | Yes | Yes | -15.49  (<0.05) | 1.09  (<0.05) | 17.7 | -15.50  (<0.05) | 1.07  (<0.05) | 18.6 |
| Yes | Yes | Yes | -- | -- | -16.17  (<0.05) | 1.22  (<0.05) | 39.8 | -15.83  (<0.05) | 1.10  (<0.05) | 42.4 |
| Yes | -- | Yes | -- | -- | -16.26  (<0.05) | 1.08  (<0.05) | -1.9 | -15.20  (<0.05) | 0.84  (<0.05) | 42.3 |
| -- | Yes | Yes | -- | -- | -14.45  (<0.05) | 0.82  (<0.05) | 73.9 | -14.18  (<0.05) | 0.72  (<0.05) | 73.4 |

* indicates a significant improvement in model fit compared to the referent all-elements model.
