# Supplementary material for: Evaluation of the effectiveness of the California mosquito-borne virus surveillance & response plan, 2009–2018
Source: PLoS Negl Trop Dis. 2022 May 9;16(5):e0010375. doi: 10.1371/journal.pntd.0010375 (PMC9119623; doi:10.1371/journal.pntd.0010375)
Supplement: S4 Table — Lower AIC values indicate better fit. (DOCX) [file pntd.0010375.s004.docx]

**S4 Table**: Comparison of coefficients from models to predict human WNV disease occurrence, CA, 2009-2018 by Response Plan and vector index, ranked by Akaike Information Criterion (AIC). Lower AIC values indicate better fit.

|  | ***Cx. pipiens* complex models** | | | ***Cx. tarsalis* models** | | |
| --- | --- | --- | --- | --- | --- | --- |
| **Predictor** | **Intercept (p-value)** | **Slope**  **(p-value)** | **ΔAIC** | **Intercept (p-value)** | **Slope**  **(p-value)** | **ΔAIC** |
| *Overall risk level* | *-17.22 (<0.05)* | *1.25 (<0.05)* | *(referent)* | *-16.46 (<0.05)* | *1.08*  *(<0.05)* | *(referent)* |
| Vector Index | -15.91 (<0.05) | 0.002 (<0.05) | 1,829 | -13.09 (<0.05) | 0.001 (<0.05) | 1,552 |
